# Supplementary material for: Are carbon emissions trading and green financial instruments synergistic? -Comprehensive quantitative research based on content analysis
Source: PLoS One. 2024 Mar 7;19(3):e0298601. doi: 10.1371/journal.pone.0298601 (PMC10919601; doi:10.1371/journal.pone.0298601)
Supplement: S1 Data — (ZIP) [file pone.0298601.s001.zip › Data/地级市Z_F工作报告.zip!/╘╞─╧/╟·╛╕╩╨/└┤╫╘╣1⁄2╓┌║┼í╛┬φ┐╦╩2╛▌═°í┐_2014.docx]

各位代表：

现在，我代表市人民政府，向大会报告工作，请予审议，并请市政协委员提出意见。

一、2013年工作回顾

2013年，是本届政府的开局之年，是困难较多、矛盾突出的一年，也是经济社会发展取得显著成效的一年。在省委、省政府和市委的领导下，在市人大、市政协的有效监督和大力支持下，我们按照“一年打基础、两年见成效、三年新跨越、率先建小康”的总体要求，以敢于担当的勇气直面矛盾，以科学务实的举措破解难题，着力稳增长、调结构、增投资、强基础、优环境、惠民生，保持了经济社会平稳健康发展的态势。完成市内生产总值1583.9亿元，同比增长13.1%；规模以上固定资产投资1020.8亿元，同比增长23.7%；财政总收入374.3亿元，同比增长9.2%；地方公共财政预算收入121.5亿元，同比增长17%；社会消费品零售总额378.3亿元，同比增长14.1%；城镇居民人均可支配收入24261元，同比增长12.2%；农民人均纯收入6861元，同比增长15.3%。除社会消费品零售总额、城镇居民人均可支配收入两项指标外，其他主要指标均完成或超额完成市四届人大一次会议确定的目标任务。

过去的一年，我们主要抓了以下6个方面的工作：

（一）努力保持经济平稳增长

面对宏观经济下行压力加大、经济增长乏力的严峻形势，出台了促进经济平稳较快发展的34条措施，及时帮助企业解决生产经营、项目建设、技术改造等方面的困难和问题。落实企业技改、促销奖励等扶持资金2.2亿元，支持企业通过发行债券等方式直接融资10亿元，促成74户企业与金融机构达成了309.8亿元的融资意向。改革行政审批制度，取消、下放和调整了120项行政审批事项；优化再造审批流程，实行投资项目并联审批，行政审批事项法定时限压缩了三分之一以上。争取上级项目补助资金22亿元，带动民间投资662亿元，推动787个项目开工建设、679个项目竣工投产，确保了投资快速增长。落实促进消费增长的政策措施，消费市场繁荣发展。建立健全招商引资优惠政策和考核奖惩制度，充分发挥产业招商组和招商分局的作用，实施招商引资项目369个，同比增长44.1%；实际到位市外国内资金371.9亿元，同比增长33.9%；实际利用外资5766.9万美元，同比增长46%，引资额创历史新高。实现外贸进出口总额3.7亿美元，同比增长30.2%。

（二）全力加快重大基础设施建设

面对财政收支矛盾突出、基础设施建设筹资困难等问题，加大资本运作力度，对市级部分投融资公司进行整合，组建了曲靖市发展投资集团有限公司，筹集资金25.9亿元，有效缓解了建设投入不足的难题。打响综合交通基础设施建设三年攻坚战，投入15.5亿元建设资金，确保了南北大通道会泽段、普宣高速公路建设顺利推进，西石“一改高”、国道213线江底至钢厂段开工建设，宣曲、曲靖东南过境线等5条高速公路前期工作进展顺利，新建和改造农村公路3100公里。水利建设力度加大，洞上、红石岩等20件骨干水源工程和莲花田、王家冲等320件病险水库除险加固工程快速推进，阿岗、车马碧水库项目建议书已报国家部委审查。建成“五小”水利工程5.9万件，解决了46.7万人的饮水安全问题。改造中低产田地51.6万亩。完善“麒沾马”一体化电网规划，建成6个重点输变电工程，改造农网1546.9公里、城网226公里。在6个县城实现了4G网络覆盖，信息网络覆盖面进一步扩大。

（三）着力推动产业持续健康发展

启动曲靖市产业发展布局规划，完成工业园区规划修编、重化工循环经济发展方案编制和高原特色农业、昆曲绿色经济示范带等规划，制定了产业发展指导目录，进一步明确了产业发展定位、空间布局和产业体系。工业经济平稳增长，实现工业增加值736.4亿元，同比增长14.9%。实施了185个技改项目，推动能源、冶金、建材等产业改造升级，云维、驰宏等企业产业链进一步延伸，曲靖钢铁集团顺利通过工信部钢铁行业规范条件公告。泽鑫铝业一期等26个项目建成投产，会泽烟厂30万箱卷烟技改等53个续建项目加快推进，红云红河曲靖卷烟厂仓储物流等37个项目开工建设。发挥企业创新主体作用，新增高新技术企业8户、创新型试点企业3户，认定科技型中小企业32户、省级企业技术中心3户。商标战略取得新突破，新增中国驰名商标3个。民营经济增加值占全市生产总值的45.3%，比上年提高了2个百分点。园区建设取得新进展，曲靖煤化工园区创建为国家级新型工业化产业示范基地，全市工业园区产值占工业总产值的55%，圆满实现了“三年倍增”目标。农业经济稳步发展，实现农业增加值289.2亿元，同比增长7%。重点扶持5个产粮大县扩大商品粮基地建设，粮食总产达318.2万吨，实现了“十一连增”。实现肉类总产182.3万吨，同比增长9.1%。收购烟叶404.7万担，实现收购产值52.3亿元。加快发展高原特色农业，在融资方式、用地扶持和发展模式等方面先行先试，启动了38个农业庄园建设，扶持珠江种业园等10个园区和双友牧业、东恒等10强龙头企业发展壮大，新增农民专业合作示范社46个，农产品加工转化率达54%。实施服务业发展“五年行动计划”，第三产业快速发展，实现增加值456.3亿元，同比增长12.4%。稳步推进60个商贸物流项目，新建、改造了100个农家店，城乡市场体系不断完善。完成了145个村邮站建设。现代金融服务业加快发展，新增2户证券类机构、7户小额贷款公司、13户股权投资类企业，银行业金融机构存贷款余额分别达1605亿元和1018亿元。旅游业发展步伐加快，三国历史文化等一批旅游项目稳步推进，实现旅游综合收入75.7亿元，同比增长18.8%。

（四）积极推进城乡协调发展

建立完善市规划领导小组、规划委员会、规划局和规划分局四级决策管理体系，集中统一珠江源大城市规划区内的城市规划审批权，促进规划管理的规范化、法制化。推进珠江源大城市总体规划、专项规划、控制性详细规划修编工作，开展控制区内1450平方公里的地形图补测绘等基础工作，启动中心城区地下管线普查。累计完成了11974个村庄规划编制。坚持以“四城联创”为统领，启动中心城区人居环境提升行动，拆临拆违、户外广告整治等工程全面展开，“两江”治理实现全线截污。县城建设步伐加快，启动了一批基础设施、城镇上山和旧城改造项目，县以上建成区面积达231平方公里。20个省级特色小城镇、11个市级重点小城镇建设顺利推进。城市区划调整工作有序推进，沾益马龙撤县设区、罗平撤县设市已上报国务院审批，完成了15个乡镇改设33个街道区划调整工作，19.3万农业人口转为城镇居民，全市城镇化率达41.6%。启动新一轮扶贫开发工作，实施 4个整乡推进、45个山区连片综合扶贫开发试点项目建设，完成了173个新农村重点村、550个整村推进重点村建设，解决了20万贫困人口的脱贫问题。

（五）认真办好社会关注的民生实事

财政用于民生的投入达223.9亿元，占地方公共预算支出的75.3%。新增城镇就业3.2万人，城镇登记失业率为3.3%。农村劳动力新增转移就业11.9万人。各项社会保险参保人数累计达476.1万人次。新农合政策范围内住院补偿比例提高到78.3%，减免补偿费用16.4亿元。率先在全省实施城乡居民大病医疗保险。为9.5万城镇低收入者、42万农村贫困人口发放最低生活保障金7亿元。完成7所农村敬老院改扩建。开工建设保障性住房2.46万套，实施农村危房改造及地震安居工程1.34万户。出台了关于加快推进教育事业改革发展的意见，对县城每新建一所中小学分别补助1500万元和1000万元，对新建标准化幼儿园按每班25万元的标准给予补助。启动市民中迁建和曲一中、市二小创建分校工作，新建、改扩建中小学228所、幼儿园232所，排除中小学D级危房33.9万平方米。学前教育快速发展，义务教育巩固提高，高中教育水平位居全省前列。启动了市中医院和市妇幼保健院搬迁建设，新开工1个县级医院，完成了247个村卫生室、24 个乡镇卫生院建设，启动了100个村卫生室建设。全面推行国家基本药物制度，实现基层医疗机构国家基本药物全覆盖。人口自然增长率控制在6.42‰以内。投入21亿元的市“五馆一中心”主体工程按期完工，省第十四届运动会、第十届残运会和市第三届运动会筹备工作全面推进。文化惠民工程深入实施，新建了165个乡镇综合文化站、社区文化活动中心和村文化室。市委、市政府承诺办理的“10件实事”取得实效。完成营造林86.6万亩、低效林改造50万亩、陡坡地治理10.3万亩、石漠化治理24.5万亩，森林覆盖率达43%。加强重点地区、重点行业、重点企业环境风险排查，实施20项重点节能技改项目和186个重点减排项目，淘汰落后产能131.9万吨，单位生产总值能耗下降3.5%。

（六）切实抓好政府自身建设

自觉接受市人大的法律监督、工作监督和市政协的民主监督，坚持重大事项及时主动向市人大报告、与市政协专题协商制度，认真听取各民主党派、工商联及无党派人士的意见建议，145件人大代表议案、建议和306件政协提案全部办复。修订完善政府工作规则，出台重大行政决策程序规定。推进政务服务体系建设，完成审批服务141.2万件。加强公共资源交易综合监管，交易总额达212.9亿元。严格贯彻执行中央“八项规定”和省、市委的有关要求，深入开展“作风转变年”活动，严肃整治“四风”，着力解决“四难”，行政监察和审计监督力度加大，通报批评和问责处理干部298人，清理“吃空饷”人员330名，“三公经费”比上年下降9.1%，政府机关作风进一步好转。

深入推进法治曲靖建设，全社会法制意识进一步增强。完善社会舆情分析、社会稳定风险评估和多元化矛盾纠纷调处机制，开展领导干部大接访、大下访活动，突出信访问题得到妥善处理。严厉打击违法犯罪行为，社会和谐稳定局面进一步巩固。严格落实安全生产责任制，全面开展安全生产大检查和综合治理，安全生产形势总体平稳。启动食品药品监管体制改革，食品药品专项整治取得阶段性成效。修订完善应急预案，加强应急演练，应急处置能力不断提高。

妇女儿童、红十字、慈善、残疾人等事业均取得新成绩。国防动员、民族、宗教、移民开发、档案、地方志、统计调查、社会科学、知识产权保护、外事侨务、保密、地震、人防、气象、水文等工作进一步加强。

各位代表，这些成绩的取得，是省委、省政府和市委坚强领导的结果，是市人大、市政协有效监督、大力支持的结果，是全市各族人民共同努力的结果。在此，我代表市人民政府，向全市广大干部群众，向人大代表、政协委员，向各民主党派、工商联、各人民团体和社会各界人士，向驻曲人民解放军和武警部队官兵，向所有关心支持曲靖发展的同志们、朋友们，表示崇高的敬意和衷心的感谢！

在肯定成绩的同时，也要清醒地看到，我们在发展中还面临着很多困难和挑战，主要是：经济总量小，产业层次低，经济增长的内生动力不强；基础设施建设欠账较多，城乡统筹任务艰巨；生态环境还很脆弱，发展与保护的矛盾依然突出；社会治理面临一系列新问题、新挑战，安全生产形势依然严峻；少数干部缺乏工作激情，执行力、创新力不足的问题还较为突出。针对这些问题，我们将采取有力措施，认真加以解决。

二、2014年主要任务

2014年，是全面深化改革的第一年，是全面完成“十二五”规划目标至关重要的一年。虽然世界经济新的增长动力源尚不明朗，国内经济运行存在下行压力，但也要看到，随着我国全面深化各项改革的红利不断释放，国家更加重视西部地区发展，以及云南重要桥头堡建设和沿边金融综合改革试验区、滇中城市经济圈等战略部署的深入推进，将为我们创造更好地经济社会发展环境。

今年政府工作的总体要求是：以邓小平理论、“三个代表”重要思想和科学发展观为指导，深入贯彻落实党的十八大，十八届二中、三中全会，习近平总书记系列重要讲话和中央经济工作会议精神，按照省委九届七次全会和市委四届四次全会的部署，围绕在全省率先全面建成小康社会的目标，把改革创新贯穿于经济社会发展各个领域各个环节，坚持稳中求进、改革创新、积极作为、科学发展，确保全市经济持续健康发展、社会和谐稳定。

今年经济社会发展的主要预期目标建议为：市内生产总值增长11%以上，地方公共财政预算收入增长13%以上，规模以上固定资产投资增长23%以上，社会消费品零售总额增长13%以上，外贸进出口总额增长14%以上，城镇居民人均可支配收入增长11%以上，农民人均纯收入增长13%以上，居民消费价格总水平涨幅控制在3.5%以内，城镇登记失业率控制在4.3%以内，单位生产总值能耗下降3.4%，人口自然增长率控制在6.0‰以内。

完成以上目标，我们要突出抓好9个方面的重点工作：

（一）以重点领域改革为突破，激发经济社会发展活力

积极推进行政管理体制改革。推进政府机构改革。按照“精简、效能、统一”的原则，强化机构和职责整合，理顺权责关系，严控机构编制，确保财政供养人员只减不增。深化行政审批制度改革。承接好中央、省下放的审批事项，精简下放市级行政审批事项，并处理好放权与监管服务的关系；建立行政审批事项目录清单制度和动态管理机制，推进投资项目审批流程再造，建立简易审批程序和并联审批机制，完善网上审批大厅及监管机制；清理规范行政审批中介服务工作，推动中介机构与审批机构脱钩，加快建设市级中介服务管理中心。出台政府向社会组织购买服务的实施办法，加大政府购买公共服务力度。

全面深化经济体制改革。激发市场主体活力。推行负面清单管理模式，制定非公经济准入目录，推进工商注册制度便利化改革，落实民营企业“国民待遇”，鼓励民营企业与国有企业相互参股发展混合所有制经济。加快投融资体制改革。运用政府特许经营权，推动民间资本进入基础设施、基础产业、金融服务和社会事业等领域；加强公共资源交易体系建设，提高公共资源配置市场化水平。深化农村改革。全面开展农村土地承包经营权、农村集体建设用地使用权确权登记颁证，稳妥推进农村住房财产抵押、担保转让试点；建立农村产权流转交易平台，推动承包土地和林地使用权适度流转；推进“农转城”配套综合改革试点。深入推进政府和部门预决算公开，完善全口径预算管理体系，建立跨年度预算平衡机制。抓好麒麟、沾益等5个“三农”金融服务改革创新试点。

加快推进社会事业体制改革。稳妥推进事业单位分类改革，探索推动公办事业单位与主管部门理顺关系和去行政化。深化教育领域综合改革，探索公办学校校长教师交流轮岗制度，促进城乡优质教育资源均衡配置。推进公立医院改革，鼓励社会办医，探索构建以区域医疗联合体为基础的新型城乡医疗服务体系。深化文化体制改革，推进公益性文化事业单位劳动人事、收入分配和社会保障制度改革。

（二）以高原特色农业为抓手，扎实做好“三农”工作

提高粮食保障能力。落实粮食安全行政首长负责制，严格执行各项强农惠农政策，确保粮食播种面积在1000万亩以上，推广科技增粮措施，提高粮食生产能力，力争粮食总产达330万吨以上。完善市、县粮油物资储备体系，加强成品粮动态储备，完成农村10万户小粮仓建设，确保粮油物资稳定供应。

加快发展高原特色农业。深入推进昆曲绿色经济示范带建设，大力扶持家庭农场、专业大户、农民合作社、龙头企业和精品庄园等新型主体，重点培育10个省部级现代农业示范点，分层次、分产业抓好农业精品庄园建设，扶持10强农产品加工企业，建立18个种养专业合作示范社、9个农机专业合作社。按照建设全省重要商品蔬菜基地的目标，加快11个标准化蔬菜生产基地建设。推进现代烟草农业建设，发展油菜、蚕桑、魔芋、中药材等特色产业。实施百头肉牛、千只肉羊、万头生猪畜牧业“百千万”示范场建设，提高畜牧业综合生产能力。继续推动8个万吨级水产大县建设。依托康恩贝、东恒等企业，发展生物资源及食品深加工产业。完善农业技术服务体系，开展“院市”合作研发攻关。加快发展农产品冷链物流、电子商务等面向农业的现代服务业。

改善农村生产生活条件。建设农村公路2000公里以上。实施农村危房改造及地震安居工程1.5万户。新建“爱心水窖”2万个，解决40万人以上饮水安全问题。完成广播电视村村通工程12万户。改造中低产田地40万亩。新建沼气池2万口、节能改灶3万口。推进农村环境综合整治，建设美丽乡村。

加大扶贫开发力度。以乌蒙山片区、石漠化片区、特殊贫困乡村为主战场，实施精准扶贫，建立扶贫对象动态调整机制，健全专项扶贫、行业扶贫、社会扶贫协同推进机制，抓好10个整乡推进和53个行政村、50个自然村整村推进扶贫开发，解决20万贫困人口的脱贫问题。

（三）以园区建设为载体，全力推动工业扩容提质

集中力量建设工业园区。通过整合扶持资金、直接融资等方式，加快园区水、电、路、信息等生产生活配套设施建设。建立园区规划与土地储备相结合的联动机制，力争每个工业园区收储土地1000亩以上。完善园区综合服务体系，建立“一站式”审批服务平台。探索工业园区跨区域财税分享和能耗分担机制，打造一批主业突出、配套完整、各具特色的工业园区。

调优工业存量。推动煤化工、电解铝、钢铁、水泥等行业产品内部循环、企业互补循环、产业耦合循环，提高产业竞争力。推进煤炭资源整合、机械化开采和安全质量标准化建设，以精细化工、合成材料、清洁能源产品为方向，延伸煤炭产业链。鼓励有条件的载能企业与发电企业合作，支持煤化集团收购曲靖电厂，发展煤—电—铝及铝型材、铝制品精深加工产业。以曲靖钢铁集团为重点，鼓励钢铁企业与上下游企业联合，促进矿山—能源—冶金—化工—装备制造一体化发展。限制新增水泥产能，走砂石骨料、混凝土、矿渣微粉、预制构件一体化的发展模式，延伸水泥产业链。

扩大工业增量。努力增加工业投资。加快推进100个重点工业项目，推动华睿中泥煤制气等50个新开工项目建设，加快红云红河打叶复烤异地搬迁等50个续建项目建设，力争众一精细化工永固紫等30个项目竣工投产，确保工业投资达280亿元以上。大力培育新兴产业。以昆明冶研四氯化硅综合技改等项目为重点，发展光伏产业；以乔治白、新千佛等企业为重点，发展轻纺产业；推动陆良泽瑞光电LED项目尽快开工建设，引进与安费诺、新威电子等企业上下游配套的产业链项目，发展电子信息产业；积极谋划一批风电、光伏发电、生物质能发电项目，发展新能源产业。

提升企业科技创新能力。实施新一轮创新型曲靖行动计划，推进质量兴市战略，加大品牌培育力度。落实促进企业创新的税收优惠政策，争取认定省级企业技术中心2户、高新技术企业5户以上、创新型试点企业3户以上、科技型中小企业20户以上，实施100项重点技术改造、技术创新和质量提升项目，推动企业技术创新、产品创新和市场创新。

加大资金筹措和资本运作力度。搭建银政企合作平台，力争新增银行融资突破200亿元、直接融资70亿元以上。加大企业上市培育力度，推动5户以上中小微企业纳入“新三板”交易储备库。整合政府投融资平台，注入优质资源资产，提升融资和担保能力。

（四）以现代服务业为牵引，努力扩大消费需求

做强旅游业。编制乡村旅游发展规划，推进旅游与城市、文化、生态和产业融合，加大珠江源旅游品牌营销力度，重点打造罗平、会泽等旅游精品城市和精品旅游线路，抓好三国历史文化、罗平生物群国家地质公园、会泽古城等一批文化旅游项目，增强旅游对经济社会发展的拉动作用。

积极发展金融服务业。支持具备条件的民间资本依法发起设立银行等金融机构，力争2户以上民营银行进入省级储备库，引进2户以上银行到曲靖设立分支机构。支持市内金融机构做大做强，鼓励市商业银行跨区域经营。设立股权投资政府引导基金，扶持发展3户以上股权类投资企业。推进金融产业示范园建设，增强金融业态聚集发展效应，提升金融服务的普惠性。加强典当行、寄售行、小额贷款公司监管，全面开展信用体系建设，构建良好的金融生态环境。

加快发展现代物流业。完善现代物流业发展规划，培育一批集运输、仓储、配送、信息、金融服务等为一体的现代物流企业，推动林安（曲靖）泛亚现代物流产业园尽快开工建设。加快沾益珠江源、马龙通泉等物流中心建设，改造提升宣威凤凰山、富源羊尾哨、师宗货站等铁路站点。支持邮政普遍服务设施建设。开展高铁经济、临空经济前期研究，积极发展高速公路、铁路、航空相互衔接的多式联运。

提升商贸服务业发展水平。继续推进商贸物流集聚区建设，改造提升大型农产品交易市场，建设、改造10个乡镇农（集）贸市场和一批城区菜市场，完成100个农家店信息化改造。大力培育具有国际竞争力的出口产品，支持有条件的企业走出去发展，鼓励企业建设面向行业和消费者的电子商务平台。加快发展面向生产的教育培训、信息服务和中介服务业。培育健康养老、家政服务、特色医疗和文化体育休闲等消费热点，促进新型电子产品、智能家电、节能汽车等热点消费。完善储备体系，加强重要商品产运销衔接，建立健全经营异常企业名录和黑名单制度，强化市场监管，营造良好消费环境。

（五）以投资拉动为引擎，切实增强经济发展后劲

确保投资稳定增长。完善项目推进工作机制，加大项目前期经费投入，狠抓项目落地，确保固定资产投资达1260亿元以上。建立市直部门、县（市、区）争取项目和资金考核激励机制，争取上级更多的支持。采取政府采购、贷款贴息等措施，落实税收减免政策，增强企业投资意愿和信心。健全招商项目跟踪服务制度，探索建立投资环境量化评价体系，依托工业园区，推动产业招商、园区招商、以商招商、驻点招商向纵深发展，承接好东部地区产业转移，力争引进市外国内到位资金450亿元，实际利用外资8000万美元。

推进综合交通基础设施建设。完成曲靖现代综合交通体系规划编制，全力推进综合交通三年攻坚，力争用5年左右时间，实现县县通高速目标，推动曲靖由重要交通通道向重要交通枢纽转变。加快南北大通道会泽段、普宣高速、西石“一改高”、国道213线江底至钢厂段建设，全面完成沾会、江召、兴富高速公路和国道320线、326线改造提升项目前期工作，启动宣曲高速、曲靖东南过境线项目建设。支持沪昆客运专线建设，积极争取渝昆铁路在曲靖设立站点。加强公路管护，提升路况质量。

推进“兴水强市”战略。加快推进20件在建骨干水源工程、320件病险水库除险加固工程，开工建设宣威沈家河等6件重点水源工程，新开工100件以上小（二）型病险水库除险加固工程。争取阿岗、车马碧水库尽快通过国家审批，争取龙潭水库通过省级审批，扎实做好黑滩河水库项目前期工作。完成干支渠防渗150公里，实施水土流失综合治理400平方公里，抓好富源块泽河等11条中小河流治理工程。

推进信息和电网基础设施建设。积极推进4G网络建设，提高市县乡三级网络覆盖率。推进“两化融合”，提高企业信息化水平。推进电子政务外网建设和应用，确保网络信息和通信安全。开工、续建35千伏以上输变电工程35个，抓好1246个城乡电网改造项目，提升电网安全保障能力。

（六）以人的城镇化为核心，统筹推进城乡发展

优化城镇规划布局。完善新型城镇化规划体系，构建以珠江源大城市为核心、县城为重点、小城镇为骨干、中心村为补充的城镇发展格局。开展城市空间体系规划，建立空间基础数字系统。完善珠江源大城市规划体系，加快“麒沾马”同城化步伐，促进区域基础设施、公共服务设施、生态环境共建共享，提升珠江源大城市品质。加快沾益马龙撤县设区、罗平撤县设市进程，支持宣威、罗平建设区域性中心城市。注重功能与特色相统一，打造一批交通枢纽型、工矿园区型、商贸集散型、民族文化型、休闲旅游型特色小城镇。统筹异地搬迁、库区移民、矿村共建、扶贫开发等项目，加强传统村落保护和特色民居建设，加快建设一批人口集中、设施齐全、商品集散的中心村。

提升城乡人居环境。深入推进“四城联创”，做好国家园林城市创建申报工作，力争成功创建国家卫生城市。实施中心城区人居环境提升行动，全面完成中心城区“五纵五横”道路改造提升等工程，启动5个人工湖建设，完成“两江”治理后期工作。统筹老城区、新城区、产业园区协调发展，稳步推进旧城改造，加快推进“城镇上山”，严格按规划配建市政基础设施和公共服务设施，力争每个县（市、区）建设2个以上城市综合体。抓好20个省级特色小城镇、11个市级重点小城镇建设，加快31个重点小城镇“一水两污”设施建设。推进以人为核心的城镇化，强化权益保障，做好农业人口转户进城工作。

提高城镇管理水平。加快曲靖城市管理条例修订工作，推进城市综合执法能力建设，提高城镇法制化、科学化、人性化管理水平。健全规划管理体制机制，建立规划管理信息平台，强化规划审批监管，确保规划的严肃性和权威性。推进拆临拆违和广告设置规范等工程，加大交通秩序、施工扬尘、小街小巷综合整治力度，提升城镇便民利民水平和服务能力。

（七）以生态文明建设为前提，推动经济社会可持续发展

加强生态建设和环境保护。严格落实资源有偿使用、生态补偿、生态环境损害问责与赔偿制度，加大环保、林政执法力度,从源头上控制环境污染和生态破坏。开展珠江源大城市林业生态圈规划，实施三年城乡绿化攻坚行动，启动新一轮退耕还林工程，继续实施天然林保护、石漠化治理、农村能源建设等重点工程，发展速生丰产林20万亩、木本油料基地20万亩，实施陡坡地治理10万亩、低效林改造40万亩。加大地质灾害区、生态脆弱区和矿山环境恢复治理力度，抓好饮用水源周边环境污染专项治理，积极开展重金属污染防治。抓好中心城区PM2.5等空气质量指标监测工作。

落实节能减排措施。坚持绿色循环低碳发展和清洁生产，严格环境准入和节能减排标准，加大重点地区、重点行业、重点企业节能减排监管力度，坚持污染物排放许可制，开展节能量、碳排放权和排污权交易试点，积极推行环境污染第三方治理模式。加快曲靖市固体废物集中处理项目建设，有序推进工业园区污水处理厂建设。抓好合同能源管理、节能技术改造财政以奖代补项目建设，严控新上“两高”和产能过剩项目。

集约节约利用资源。加强矿产资源开发管理，推进低丘缓坡土地综合开发利用和城乡建设用地增减挂钩试点，加大对闲置土地、批而未用土地的清查和处置力度。推进水资源价格改革，加大水资源循环利用和高效节水示范项目建设力度。

（八）以改善民生为归宿，不断提升社会建设水平

千方百计增加就业。统筹做好高校毕业生和城镇就业困难人员等重点人群就业工作，加大“贷免扶补”、小额担保贷款和劳动密集型小微企业贷款力度，提供就业岗位4万个，新增城镇就业 2.6万人，扶持1.6万人自主创业，新增转移输出农村劳动力8万人。深入实施“城乡居民收入倍增计划”，落实提高企业职工养老、工伤和失业保险待遇等政策，多渠道增加农民收入。

健全社会保障体系。扩大社保参保缴费覆盖面，力争主要险种覆盖率均保持在95%以上。落实城乡养老保险制度衔接办法，推进城乡居民养老保险基金市级管理。完善社会救助体系，整合各类救助资源，使帮困救助工作更加规范化、系统化、制度化。落实社会救助、社会保障标准与物价上涨挂钩机制，确保城乡低保应保尽保。发挥慈善事业在社会关爱中的重要作用。加快社会养老服务体系建设，新建7所农村敬老院、30个社区居家养老服务中心，鼓励发展老年服务产业。完善住房公积金管理制度。健全保障性住房建设、供给、分配制度，新开工2.47万套保障性住房。

推进教育均衡发展。继续推进中小学校舍安全工程，排除D级危房20万平方米。落实标准化幼儿园和县城中小学新建补助政策，抓好市民中迁建和曲一中、市二小创建分校工作，新建、改扩建86所幼儿园。推进中小学信息化、标准化建设。大力发展学前教育，巩固提高义务教育水平，稳步提高普通高中教育水平，促进职业教育和高等教育健康发展。

提升公共卫生服务水平。加快市中医院、市妇幼保健院搬迁、市全科医生临床培养基地建设，完成1个县级医院、23个乡镇卫生院和100个村卫生室改扩建，推动城市社区卫生服务体系建设。完善基层医疗卫生机构补偿机制，提高基本药物采购配送效率。完善大病补充商业保险制度，提高新农合筹资标准和规范化管理水平。继续实施乡村医生3年培养计划。落实国家计划生育政策，促进人口均衡发展。

加快公共文化体育服务体系建设。完善公共文化体育设施，实施文化惠民工程，加大文化艺术精品创作力度，丰富群众文化生活。支持文化创意、演艺娱乐、出版传媒等文化产业与相关产业融合发展。继续推进七彩云南全民健身、文化信息资源共享等工程，加强重点文化遗产保护、传承和利用。抓好市“五馆一中心”装修布展和文化体育公园运营管理，全力承办好省第十四届运动会和第十届残运会，筹办好市第三届运动会。

统筹做好外事侨务、档案、保密、地方志、移民开发、社会科学等工作，推进红十字、残疾人等事业发展。

改进社会治理方式。加强法治曲靖建设，运用法治思维和法治方式化解社会矛盾。强化社会组织培育引导和规范管理，充分发挥社会组织在社会治理中的作用。发挥领导干部和公职人员的示范带头作用，建立健全志愿者服务体系，充分发挥志愿者队伍在公益服务等方面的积极作用。落实安全生产属地管理责任、部门监管责任和企业主体责任，探索安全生产清单式管理模式，建立隐患排查治理体系和安全防控体系，深入开展“打非治违”和安全隐患专项整治，坚决遏制重特大事故的发生，促进安全生产形势持续稳定好转。更加注重食品药品生产、流通、消费全程监管，提高精细化、精准化、全覆盖的食品药品安全监管水平。加强地震等防灾减灾能力建设，完善应急管理体系，提高应急处置能力。健全重大决策社会稳定风险评估和矛盾纠纷排查调处机制，完善网上信访制度，推行部门集中联合接访和市县乡三级视频接访，打造阳光信访。完善社会治安立体化防控、网格化管理体系，健全网络舆情分析、引导和处置机制，严厉打击各种违法犯罪行为。

加强国防后备力量建设，积极做好拥军优属、国防动员、民兵预备役和人民防空工作。贯彻落实党的民族政策，推进民族团结进步边疆繁荣稳定示范区建设。贯彻落实党的宗教工作方针，发挥宗教界人士和信教群众在促进经济社会发展中的积极作用。

（九）以人民满意为目标，全面加强政府自身建设

认真履行政府职能。正确处理政府和市场的关系，积极推进政府职能转变，充分履行宏观调控、市场监管、公共服务、社会管理、环境保护职能，充分发挥市场在资源配置中的决定性作用，切实把政府工作重心转移到创造良好发展环境、提供优质公共服务和维护社会公平正义上来，进一步激发市场和社会主体的创造活力，增强经济发展的内生动力。

严格依法行政。自觉接受市人大的法律监督、工作监督和市政协的民主监督，接受人民群众和新闻舆论的广泛监督。认真办理人大代表议案、建议和政协提案。完善科学民主决策机制，坚持公众参与、专家咨询、风险评估、合法性审查和集体讨论决定的重大事项决策程序。落实行政执法责任，严厉查处各种乱检查、乱收费、乱罚款等滥用行政执法权的行为。

坚持勤政为民。把敢于担当作为政府工作的座右铭，把狠抓落实作为政府工作的基本职责，用抓项目的理念推进工作，以目标引领，以时间倒逼，以责任问效，不断推动工作实现新突破。完善社会评议制度，建立完善政府绩效管理和考核方式，不断提高行政效能。开展好党的群众路线教育实践活动，以真挚的感情多为群众办实事，以满腔的热情多为企业解难题，努力营造风清气正的发展环境。

加强廉政建设。自觉遵守中央“八项规定”和省、市委相关规定，坚决整治“四风”，发扬艰苦奋斗的优良传统，厉行节约，反对浪费，严控“三公经费”，以良好的政风推动各项工作。全面落实党风廉政建设责任制，认真履行“一岗双责”，切实抓好政府系统反腐倡廉工作，强化行政监察和审计监督，加强对重点领域、关键环节、重要岗位权力运行的监督制约，严肃查处各类违纪违法案件，做到干部清正、政府清廉。

各位代表！百舸争流，千帆竞发，时不我待。让我们高举中国特色社会主义伟大旗帜，在省委、省政府和市委的坚强领导下，以更加饱满的工作激情、更加务实的工作作风，抢抓新机遇，争创新优势，实现新发展，为在全省率先全面建成小康社会而努力奋斗！
